# Real-World Safety Profile of Adjuvant Abemaciclib in HR+/HER2− Early Breast Cancer

**DOI:** 10.3390/cancers18162543

**Published:** 2026-08-07

**Authors:** Maria Pimenta Macedo, Isabel Dionísio Sousa, Nuno Teixeira Tavares

**Affiliations:** 1 Faculty of Medicine, University of Porto, 4200-319 Porto, Portugal; 2 Oncology Department, São João University Hospital, 4200-319 Porto, Portugalnuno.tavares@ulssjoao.min-saude.pt (N.T.T.)

**Keywords:** Abemaciclib, CDK4/6 inhibitors, early breast cancer, adverse events, dose reduction, treatment interruption, real-world data

## Abstract

**Background/Objectives:** Treatment with adjuvant abemaciclib plus endocrine therapy (ET) has been demonstrated to improve outcomes in patients with HR+/HER2- early breast cancer at high risk of recurrence. However, real-world data regarding abemaciclib tolerability and treatment maintenance remain limited. In this study we describe the occurrence of adverse events (AEs) and their management through dose adjustments—dose reductions and temporary treatment interruptions—reflecting strategies that maximize compliance. **Methods**: We conducted a retrospective cohort study at the Oncology Department of ULS São João, in Porto, Portugal. Inclusion criteria comprised patients with HR+/HER2- early breast cancer treated, for at least three months, with abemaciclib, between January 2022 and January 2026. Clinical data were collected from electronic medical records and were analyzed using descriptive statistics, with continuous variables reported as median (IQR) and categorical variables as frequencies and percentages. **Results:** Fifty-six patients were included in this cohort. With the median follow-up of 14.5 months, 98.2% of patients experienced at least one AE. Most common AEs were gastrointestinal and hematological and occurred within the first 3 months of treatment. Dose reductions occurred in 46.4% of patients and temporary treatment interruptions in 25% of patients. However, no permanent treatment discontinuations were reported. **Conclusions:** In this cohort, AEs associated with adjuvant abemaciclib were common but generally low-grade and manageable. Early recognition of AEs allows appropriate dose adjustments and supportive measures, which support treatment maintenance. These findings highlight the importance of close clinical monitoring and patient counseling to enhance treatment compliance.

## Simple Summary

Breast cancer remains one of the most prevalent malignancies worldwide, with a subset of patients presenting high risk of recurrence despite standard treatment. Abemaciclib is an oral, selective, CDK4/6 inhibitor that has recently been introduced in the adjuvant setting, in combination with hormone therapy, to reduce the risk of recurrence. However, the safety profile of abemaciclib in real-world clinical practice has not yet been fully elucidated. Therefore, in this study, we evaluated patients treated with abemaciclib at a Portuguese oncology center in order to characterize the occurrence of adverse events and their management. We found that most adverse events were mild and manageable, concluding that, with careful clinical monitoring and timely dose adjustments, permanent discontinuations could be avoided, enhancing treatment maintenance.

## 1. Introduction

Breast cancer is characterized by significant heterogeneity and remains one of the leading causes of malignancy-related deaths in women around the world [1]. According to GLOBOCAN 2022 estimates of cancer incidence, produced by the International Agency for Research on Cancer, about 2.29 million new cases of breast cancer were diagnosed in 2022 worldwide, being the second most commonly diagnosed cancer, only surpassed by lung cancer [2]. According to The Global Cancer Observatory (GCO), 8954 people were diagnosed with breast cancer in Portugal in 2022, and 2211 died [3]. Even though the mortality rate has decreased in recent years in Western populations, breast cancer is still the leading cause of cancer-related deaths for women worldwide [4].

Breast cancer can be classified into three different subtypes based on the immunohistochemistry assessment of hormone receptor (HR) status, including estrogen receptor (ER) and progesterone receptor (PR) status and human epidermal growth factor receptor-2 (HER2) [1,5]. There are HR-positive (HR+)/HER2-negative (HER2−) tumors, HR+/HER2-positive (HER+) tumors, and triple-negative tumors (with no expression of ER, PR and HER2).

In this study, we focus on HR+/HER2− tumors that account for approximately 70% of all breast cancers [6]. At the time of diagnosis, the disease is localized to the breast and regional lymph nodes (LN)—early-stage breast cancer—in 94–97% of patients. The standard treatment for HR+/HER2− breast cancer varies with recurrence risk but includes surgery, radiotherapy and systemic therapies such as chemotherapy (neoadjuvant or adjuvant), adjuvant endocrine therapy (ET) and, more recently, targeted therapies. ET is the primary adjuvant therapy and includes aromatase inhibitors and/or antiestrogens, with or without ovarian function suppression. It has been associated with a significant reduction in the risk of recurrence and death. However, despite its proven efficacy [7], 10–30% of patients with early-stage breast cancer (EBC) will develop disease recurrence due to ET resistance [8,9].

In patients with high-risk clinical and pathological features, the risk of recurrence is increased, so it is crucial to optimize adjuvant therapy to prevent early recurrence [10]. In recent years, targeted therapies, such as cyclin-dependent kinases (CDK) 4 and 6 inhibitors, including abemaciclib, have been added to guidelines, as a therapeutic option [11]. This therapeutic class blocks tumor cell progression from the G1 phase to the S phase of the cell cycle, inhibiting tumoral cell proliferation [12]. Abemaciclib has a greater selectivity for CDK4, compared to CDK6, which plays a critical role in hematopoiesis. This has an impact on its toxicity profile [13].

Based on the results of the monarchE trial, the addition of two years of adjuvant abemaciclib to ET showed an improvement in invasive disease-free survival (IDFS) and distant relapse-free survival (DRFS) in patients with HR+, HER2− early breast cancer at high risk of recurrence. Recent data, at a median follow-up of 76.2 months, demonstrated that abemaciclib plus ET resulted in a 15.8% lower risk of death than ET alone (hazard ratio 0.842, 95% confidence interval (CI) 0.722–0.981, *p* = 0.027), meeting the prespecified boundary for significance. The 7-year Overall Survival (OS) was 86.8% with abemaciclib plus ET and 85.0% with ET alone (absolute difference, 1.8%). OS benefit was consistent across prespecified subgroups. Sustained improvement was demonstrated in IDFS and DRFS (hazard ratio 0.734, 95% CI 0.657–0.820 and hazard ratio 0.746, 95% CI 0.662–0.840, respectively), as seven-year IDFS was 77.4% with abemaciclib plus ET and 70.9% with ET (absolute difference, 6.5%) and 7-year DRFS were 80.0% and 74.9%, respectively (absolute difference, 5.1%). Safety data compiled over 7 years of follow-up did not raise any concerns of delayed toxicities [14].

In monarchE, high-risk disease was defined as the presence of at least four positive pathologic axillary lymph nodes (pALNs), or one to three pALNs combined with at least one additional high-risk feature of either histological grade 3 disease, tumor size ≥ 5 cm or Ki-67 ≥ 20% [15]. In this context, abemaciclib is now part of adjuvant treatment for patients with these characteristics, being orally administered for two years or until meeting criteria for discontinuation. Nevertheless, the addition of abemaciclib to the standard-of-care ET in patients with HR+, HER2−, node-positive, high-risk EBC, raises new challenges related to treatment tolerability and long-term persistence [16].

Therefore, we conducted a retrospective, observational cohort study, involving patients followed at the Oncology Department of ULS São João, to evaluate real-world evidence regarding safety and treatment tolerability—particularly with respect to dose modifications and interruptions or discontinuations attributable to adverse events (AEs)—within the context of routine clinical practice, as clinical trial populations may not fully reflect real-world tolerability profiles. Such data are essential to inform optimization of management strategies and to support sustained therapeutic compliance.

## 2. Materials and Methods

### 2.1. Study Design and Setting

This retrospective cohort study included patients with HR+/HER2− high-risk EBC, treated with adjuvant abemaciclib at the Oncology Department of ULS São João, in Porto, Portugal, between January 2022 and 31 January 2026.

Clinical data were collected retrospectively from electronic medical records, including demographic information, clinical characteristics and previous treatments. The variables included in the database are described in Section 3.

This study followed the Strengthening the Reporting of Observational Studies in Epidemiology (STROBE) [17] and was conducted in compliance with the Declaration of Helsinki [18]. It was approved by the Institutional Ethics Committee of the hospital (Protocol 150-22). All data were anonymized to ensure patient confidentiality; therefore, informed consent was not required.

### 2.2. Inclusion/Exclusion Criteria

Eligible patients were adults diagnosed with HR+/HER2− node-positive, high-risk EBC [high-risk criteria according to monarchE (≥4 positive axillary lymph nodes or 1–3 positive axillary lymph nodes with tumor size ≥ 5 cm and/or histologic grade 3)], who initiated adjuvant abemaciclib. HR positivity and HER2 negativity were defined in accordance with the American Society of Clinical Oncology and the College of American Pathologists guidelines. Patients were excluded if abemaciclib was administered in the metastatic setting or if follow-up data were unavailable. Patients who had not completed 3 months of follow-up since treatment initiation were also excluded, as well as patients with a synchronous malignancy.

### 2.3. Endpoints

The primary endpoint was treatment maintenance, defined as the proportion of patients remaining on treatment at predefined time points. Treatment maintenance was evaluated regarding both the proportion of patients who remained on treatment and the timing of permanent discontinuation over the course of treatment.

In addition, treatment adjustments, including dose reductions and temporary treatment interruptions, were systematically recorded and analyzed as measures of tolerability.

Furthermore, we retrospectively evaluated the occurrence of AEs on clinical records, grading them according to the Common Terminology Criteria for Adverse Events version 5.0 (CTCAEv5). For each event, we recorded the time to onset, the highest CTCAEv5 grade reached, the number of affected patients and whether the event led to treatment modification (temporary interruption, dose reduction or treatment discontinuation).

### 2.4. Statistical Analysis

Statistical analyses were performed using IBM SPSS Statistics, version 30.0 (IBM Corp., Armonk, NY, USA). Baseline patient characteristics were summarized using descriptive statistics. Continuous variables were summarized as median and interquartile ranges (IQR), and categorical variables were described using frequencies and percentages.

The primary endpoint, treatment maintenance, was evaluated as the proportion of patients remaining on abemaciclib at predefined time points (3, 6, 12, and 24 months). Treatment discontinuation was defined as permanent cessation of abemaciclib for any reason.

Secondary endpoints included dose adjustments, temporary treatment interruptions, and occurrence of AEs. The time to onset of AEs, grading and time to dose reduction either to 100 mg or to 50 mg were summarized descriptively.

## 3. Results

### 3.1. Patient Population

**Figure 1:**
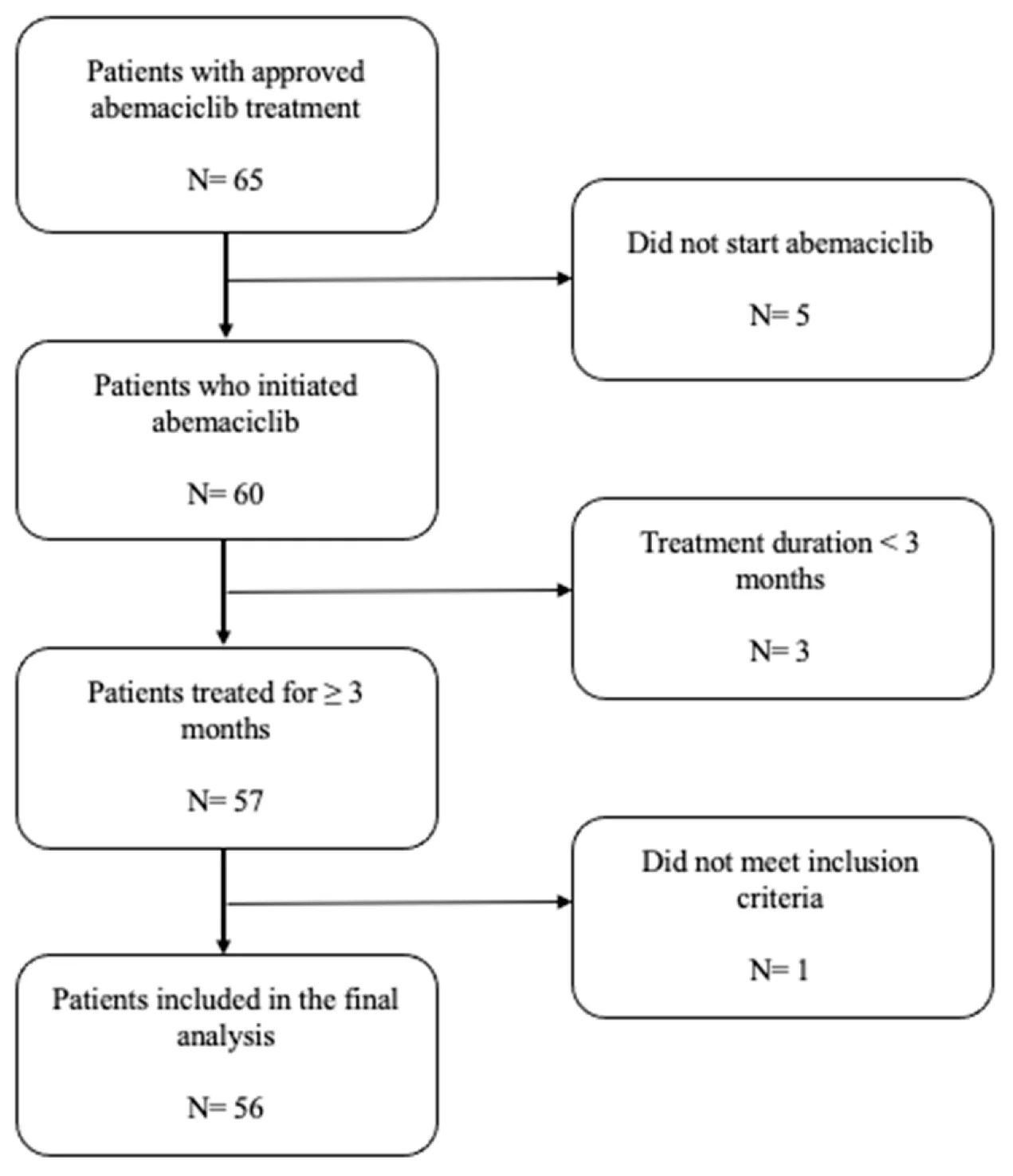
Study flowchart.

The study included 56 female patients with HR+/HER2− early-stage breast cancer, regardless of menopausal status. From January 2022 to January 2026, treatment with abemaciclib was approved for 65 patients. Figure 1 shows that only 56 of those patients were included in the final analysis. Five of them had not started treatment at data cutoff, while three patients underwent treatment for less than 3 months. One patient was excluded due to the presence of a synchronous malignancy.

The demographic, clinical, and pathological characteristics and previous systemic therapies of the cohort are summarized in Table 1. The median age at treatment initiation was 52 years, with 35.7% of patients aged <50 years, 48.2% aged 50–65 years, and 16.1% aged >65 years. Most tumors exhibited high estrogen receptor expression, with 83.9% demonstrating 91–100% positivity. Progesterone receptor expression was more heterogeneous, with 14.3% of tumors showing absence of expression. Regarding nodal involvement, 55.4% of patients had 1–3 positive LN (N1), 28.6% had 4–9 LN involved (N2), and 7.1% had >10 positive LN (N3). In total, 8.9% of patients had cytologically confirmed nodal involvement at initial diagnosis and were downstaged after neoadjuvant therapy and therefore remained eligible for adjuvant abemaciclib based on baseline nodal status. Histological grade was predominantly grade 2 (53.6%) and grade 3 (33.9%). The majority of tumors were of no special type (NST) histology (83.9%). A Ki-67 index ≥ 20% was observed in 17 patients (30.4%), while 8 patients (14.3%) had Ki-67 < 20%; Ki-67 index was not assessed in 31 patients (55.4%). Pathological tumor size was distributed as follows: 26.8% T1, 46.4% T2, and 25.0% T3. One patient (1.8%) had previously been diagnosed with left breast cancer in 2012; therefore, pT could not be accurately evaluated. Breast conservative surgery was performed in 38 patients and radical mastectomy in 17 patients. Worthwhile mentioning, one patient had a previous breast surgery due to a diagnosis of left breast cancer in 2012 that was classified as “other”. Currently she presented with axillary recurrence and underwent targeted axillary dissection. Regarding axillary interventions, 33 patients (58.9%) had axillary LN dissection, whereas 21 patients (37.5%) underwent sentinel LN biopsy. In two patients (3.6%) targeted axillary dissection was performed. Nearly half of the cohort (44.6%) received neoadjuvant chemotherapy and 50.0% received adjuvant chemotherapy, predominantly anthracycline-based regimens in both settings. Radiotherapy was administered in all patients, except for one. Endocrine therapy consisted primarily of aromatase inhibitors, including anastrozole (60.7%), exemestane (10.7%) and letrozole (1.8%), while 26.8% of patients received tamoxifen. Of the 15 patients treated with tamoxifen, 66.7% were considered as peri-menopausal and had, later on, a switch to aromatase inhibitors. All patients had an ECOG performance status (PS) of 0–1. The median follow-up duration under abemaciclib therapy was 14.5 months.

### 3.2. Treatment Maintenance

During the study period, no permanent treatment discontinuations occurred. At data cutoff, on 31 January 2026, 48 patients (85.7%) were still receiving abemaciclib, while 8 patients (14.3%) had completed the planned 2-year course of therapy, as summarized in Table 2.

### 3.3. Dose Adjustments

**Figure 2:**
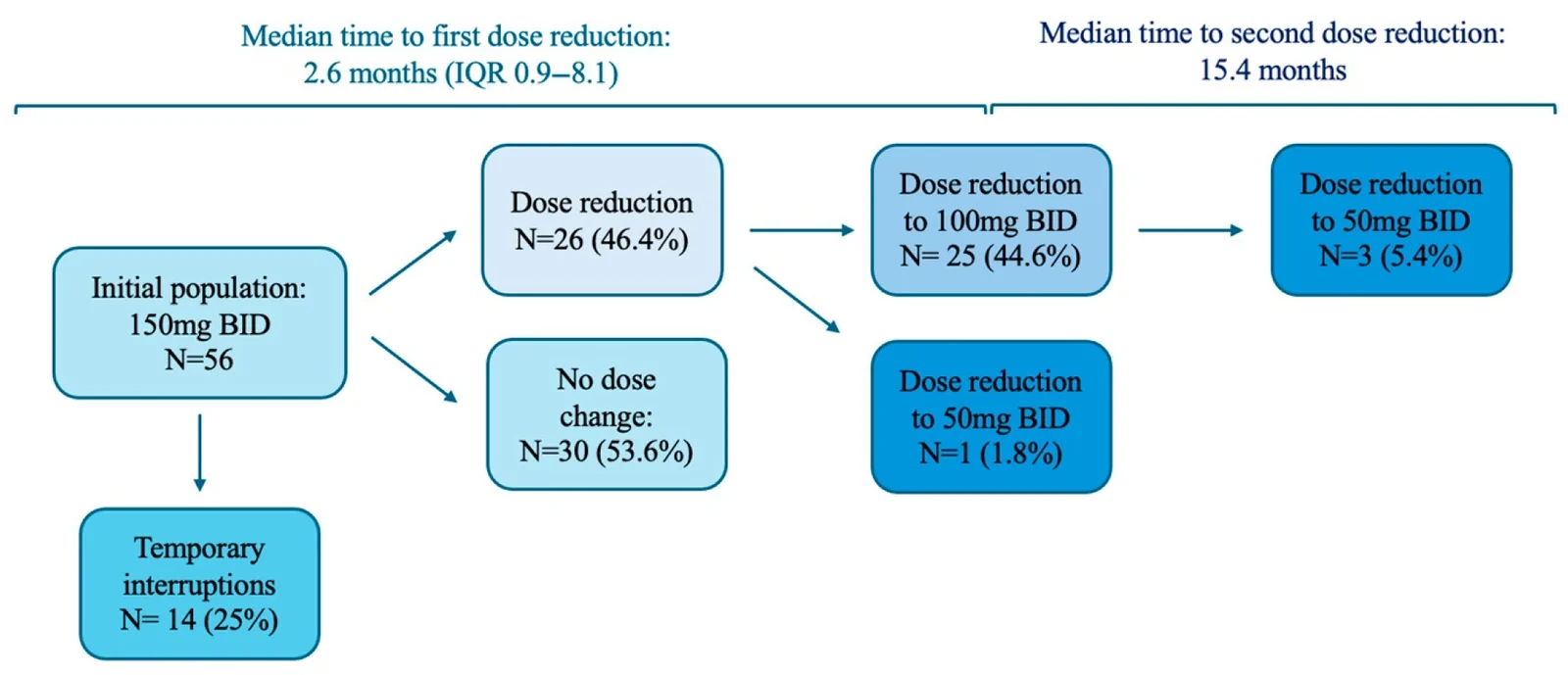
Abemaciclib safety profile.

Dose reductions were required in 26 patients (46.4%), with 25 patients (44.6%) initially reducing from the starting dose of 150 mg to 100 mg. Reduction to 50 mg occurred in four patients (7.1%). Among these, three patients (5.4%) underwent stepwise dose reduction from 150 mg to 100 mg followed by 50 mg, whereas one patient (1.8%) required direct reduction to 50 mg. The median time to first dose reduction was 2.6 months (IQR, 0.9–8.1). The median time from treatment initiation to second reduction was 15.4 months. Temporary treatment interruption due to AEs was observed in 14 patients (25.0%). The frequency and timing of dose adjustments are summarized in Figure 2.

### 3.4. Toxicity Profile

AEs observed during treatment with abemaciclib represent the leading cause of dose adjustments and temporary interruptions. During the follow-up period, 55 patients (98.2%) developed AEs of variable severity. Neutropenia was the most common AE, occurring in 50 patients (89.3%), followed by anemia in 43 patients (76.8%) and diarrhea in 40 patients (71.4%). Fatigue was reported in 31 patients (55.4%), while nausea and abdominal pain were reported in 21 and 19 patients (37.5 and 33.9%), respectively. Grade 3 toxicities were predominantly hematologic, occurring in eight patients (14.3%) due to neutropenia and in two patients (3.6%) for anemia. Only one case (1.8%) of grade 3 diarrhea was documented, and no grade 4 or 5 events were observed.

As shown in Table 3, most AEs occurred early after treatment initiation. Gastrointestinal toxicities tended to present within the first month of therapy, with nausea occurring at a median of 18 days (IQR, 14–107), abdominal pain at 27 days (IQR, 14–167), and diarrhea at 31 days (IQR, 18–112). Hematologic events also developed early, with neutropenia arising at a median of 28 days (IQR, 18–54) and anemia at 41 days (IQR, 27–76). Fatigue showed a slightly later onset, with a median time of 42 days (IQR, 27–112). Dose adjustments were driven by treatment-related AEs. Diarrhea was the most frequent contributor, being associated with dose reduction in 13 patients (23.2%) and treatment interruption in 3 patients (5.4%). Fatigue and abdominal pain each led to dose reduction in five patients (8.9%) and abdominal pain led to one treatment interruption. Neutropenia, nausea, and anemia led to dose reductions in three patients each (5.4%) and treatment interruption occurred in five patients (8.9%) due to neutropenia, in two patients (3.6%) due to anemia and in one patient due to nausea. As some patients experienced more than one AE contributing to dose adjustment, these categories are not mutually exclusive. There were two treatment interruptions attributed to other causes. Importantly, despite the high frequency of AEs, dose adjustments and temporary interruptions for toxicity management did not lead to any permanent treatment discontinuation.

On the other hand, Table 4 summarizes the temporal distribution of AEs, confirming that the majority of them occurred within the first three months of treatment. This early pattern was particularly evident for neutropenia, with 88% of cases arising within this period, followed by anemia (83.7%), fatigue (74.2%), diarrhea (70%), nausea (66.7%), and abdominal pain (57.9%). Events occurring beyond six months were uncommon across all toxicity categories, supporting the predominantly early onset of treatment-related toxicity. Overall, gastrointestinal and hematologic toxicities were the main drivers of dose adjustments in this cohort.

**Figure 3:**
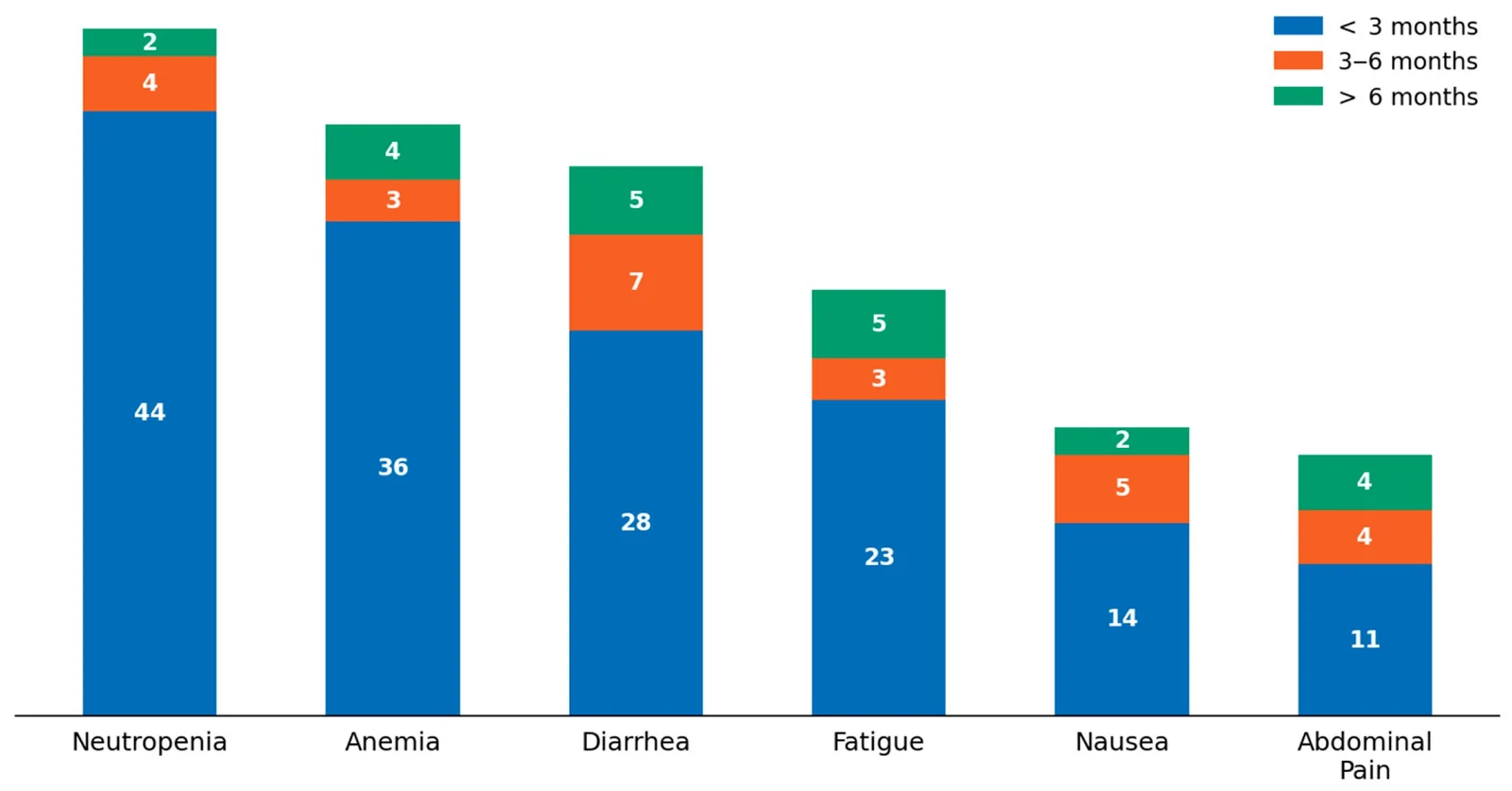
Temporal distribution of AEs onset.

Figure 3 highlights that most AEs occur early in treatment, within the first three months after abemaciclib initiation.

## 4. Discussion

In this study we evaluated a cohort of 56 patients with HR+/HER2− EBC, treated with adjuvant abemaciclib, aiming to assess treatment maintenance and dose adjustments—including dose reductions and treatment interruptions—which depicts abemaciclib’s safety profile. We observed that, in 98.2% (55 out of 56) of patients, at least one AE of any grade occurred, mostly hematological and gastrointestinal events. However, no permanent treatment discontinuations were reported.

The proportion of patients requiring dose reduction in our cohort was 46.4%, comparable to what was reported in the monarchE trial—43.7%. In the monarchE trial, 29.8% of patients had one dose reduction and 13.9% of patients had two dose reductions [16]. In this cohort, 41% of patients experienced one dose reduction but only 5.4% reported a second dose reduction, which can be influenced by the median time of follow-up of this study, since second dose reductions occur later during treatment. Dose reductions were most commonly due to diarrhea and neutropenia.

Comparing AE frequencies between the monarchE trial and this study, diarrhea was the most common event in monarchE, as it was reported in 82.2% of patients, while, in our cohort, it occurred in 71.4% of patients and was surpassed by neutropenia—89.3%—and anemia—76.8%. In monarchE, neutropenia and anemia were present in 44.6% and 22.9% of patients, respectively. The lower frequency of diarrhea observed in our cohort may reflect the fact that it is a well-recognized toxicity of abemaciclib, and antidiarrheal agents and probiotics are commonly proactively prescribed as supportive therapies. In fact, diarrhea could be underreported by patients [19]. The higher frequency of hematological toxicity in this cohort may reflect differences in baseline patient characteristics, as prior exposure to systemic treatment can be associated with an increased risk of persistent/more profound myelosuppression [20]. Fatigue and nausea were more frequent in our cohort—55.4% and 37.5%, respectively—than in monarchE—38.4% and 27.9%, respectively. Abdominal pain events were similar in both studies—33.9% in this study vs. 34% in monarchE trial. As fatigue, nausea and abdominal pain are subjective AEs, they can sometimes be underreported or misclassified, so variation in these frequencies is understandable.

The gastrointestinal effects of abemaciclib are linked to its inhibitory effect on CDK9, which plays an important role in intestinal cell proliferation. Moreover, abemaciclib exerts inhibitory activity on glycogen synthase kinase-3 beta (GSK3β) and Ca2+/calmodulin-dependent protein kinase II (CAMKII), which is involved in intestinal motility. CDK4/6 inhibition alone does not have a significant impact on intestinal cells [21,22].

It has been shown that abemaciclib is less associated with hematopoietic cytopenia, when compared to other CDK4/6 inhibitors like palbociclib and ribociclib, since it has lower inhibitory activity against CDK6, which is implicated in hematopoiesis [23,24,25]. Nevertheless, neutropenia was the most common grade 3 event in this cohort, experienced by 14.3% of patients, which is consistent with the monarchE trial findings, in which 18% of patients presented grade 3 neutropenia [9]. In this study population, there were no reported cases of febrile neutropenia. In contrast to chemotherapy, CDK4/6 inhibitor-associated neutropenia is rapidly reversible, as CDK4/6 inhibitors induce a cell-cycle arrest that is reversible following CDK4/6 dose reduction or interruption [26,27]. This rapid reversibility explains why none of the eight patients with grade 3 neutropenia required permanent discontinuation, as they were effectively managed with dose adjustments as per protocol and did not cause serious complications, namely febrile neutropenia [26,28].

In addition to assessing the frequency of AEs, it is also relevant to focus on their temporal pattern, since most AEs occurred within the first three months of abemaciclib treatment and were mainly low grade. This is consistent with other studies of abemaciclib tolerability and safety profile [9,16]. These results highlight the importance of early close monitoring, through regular clinical assessments and laboratory tests, especially if they have features that are associated with higher risk of treatment discontinuation, such as age ≥65 years or ≥4 comorbidities [29]. This allows early intervention, including dose adjustments and supportive therapies, in order to preserve patients’ quality of life.

In monarchE, in over half of permanent discontinuations there was no prior dose reduction [16]. This highlights the importance of patient counseling/awareness before starting abemaciclib treatment regarding expected AEs and their management. In clinical practice, our goal is to avoid permanent treatment discontinuations. For this purpose, it is essential that patients are closely monitored to allow early recognition of AEs, which can be managed by supportive measures, dose reductions and temporary interruptions.

Diarrhea is the most characteristic toxicity of abemaciclib, and its management is well established. Patients should be encouraged to begin antidiarrheal medication at the first sign of loose stools. If symptoms do not resolve after initiation of antidiarrheal medication, dose adjustments can improve tolerability by decreasing the severity and duration of the episodes [30]. In our study, 23.2% of patients required a dose reduction due to diarrhea, which is consistent with the monarchE trial, where 20.5% of patients required a dose reduction [16]. Other AEs should be adequately monitored and managed with supportive medications, dose reductions or temporary interruptions. A recent study showed that per protocol dose reductions did not affect adjuvant abemaciclib efficacy [29], supporting the assertion that it is a valid strategy to improve tolerability and avoid permanent discontinuation, contributing to achieving the goal of maximizing treatment compliance, in order to maintain the benefit of adjuvant abemaciclib.

Recently, the results of the TRADE trial, a prospective, single-arm, phase 2 study, were presented. This study evaluated whether dose escalation of adjuvant abemaciclib improves drug tolerability. Patients initiated abemaciclib at 50 mg BID for two weeks, then escalated to 100 mg BID for two weeks, and then escalated to the final dose level, 150 mg BID. The TRADE trial demonstrated that an early dose escalation strategy allowed more patients to reach and maintain target dosing at 12 weeks—70.8% in TRADE, compared with about 60% in monarchE. Longer follow-up is necessary to establish the impact of this strategy in the overall goal: a low rate of discontinuation. However, these results suggest that a dose escalation approach could be successful in reducing the rate of early AEs and improving treatment adherence [31].

Despite this cohort being a high-risk population in whom aromatase inhibitors should be considered, 26.8% of patients were receiving tamoxifen. Nevertheless, this proportion was lower than that reported in the monarchE trial, where 30.9% of patients were treated with tamoxifen. In our cohort, 66.7% of the patients subsequently switched to anastrozole, as they were considered perimenopausal at the time endocrine therapy was initiated. Although Abemaciclib, unlike Ribociclib, can be administered in combination with tamoxifen, treatment selection should take into account not only menopausal status but also the expected clinical benefit, toxicity profile and patient preferences.

Our study has some limitations that should be acknowledged. First, its retrospective nature introduces potential information bias, since data were collected from medical records that were not originally designed for research purposes. This can affect the classification of AEs, particularly those low-graded or subjective ones, that may be underreported or inconsistently documented. Even though anemia and neutropenia are laboratory-based AEs, fatigue, abdominal pain, nausea and low-graded diarrhea are subjective symptoms that can be inaccurately reported due to patients’ subjectivity and even clinicians’ interpretation. Selection bias may also be present, as patients receiving abemaciclib are generally selected by clinicians based on adequate performance status and clinical fitness. As a result, more fragile subgroups can be underrepresented. Moreover, our sample size (N = 56) is relatively small and may not reflect reality in all settings, as the influence of chance can be increased in our results. These factors may limit the generalizability of the tolerability profile of abemaciclib. Finally, there are some potential confounding factors that could alter our results. As previously discussed, regarding hematological toxicity, prior systemic treatment and baseline patient characteristics could influence the occurrence of AEs, since a significant proportion of patients received prior chemotherapy and radiotherapy, both of which could lead to baseline abnormalities/alterations. These confounders could not be controlled in this observational study. Additionally, transient and rapidly reversible laboratory abnormalities that are not considered abemaciclib-related toxicities were not included in the analysis.

Despite the limitations of this preliminary report, the aim of our study was to reflect real-life clinical practice and explore strategies to improve abemaciclib persistence. This was accomplished by evaluating the most common toxicities associated with treatment and how they can be managed by dose reductions or temporary interruptions. Nevertheless, our findings would benefit from an expansion of the sample size and follow-up duration as well as further validation in larger, multicenter, prospective studies, with longer follow-up.

## 5. Conclusions

In conclusion, this real-world study showed that AEs associated with adjuvant abemaciclib are common and arise early during treatment. However, these adverse events are frequently low-grade (Grade 1–2) and can be adequately managed through close clinical monitoring, helping to avoid permanent treatment discontinuations without negatively impacting patients’ quality of life. Although anticancer efficacy is traditionally the most studied endpoint, understanding treatment tolerability is equally important. As precision medicine becomes increasingly integrated into oncological treatment algorithms, it also presents the challenge of understanding how to ensure therapeutic compliance and tolerability for patients.

## Figures and Tables

**Table 1:** Demographics, baseline characteristics, and previous systemic therapies in treated population.

N (%)	Variable
56 (100)	Female sex
52.0	Age, median (years)
20 (35.7)	<50 years
27 (48.2)	50–65 years
9 (16.1)	>65 years
	ECOG performance status
52 (92.9)	0
4 (7.1)	1
	Estrogen Receptors
0	1–10%
1 (1.8)	11–50%
8 (14.3)	51–90%
47 (83.9)	91–100%
	Progesterone Receptors
8 (14.3)	0%
7 (12.5)	1–10%
9 (16.1)	11–50%
18 (32.1)	51–90%
14 (25.0)	91–100%
	Ki-67 (≥20%)
17 (30.4)	Yes
8 (14.3)	No
31 (55.4)	Missing
	Histological grade (biopsy)
5 (8.9)	Grade 1
30 (53.6)	Grade 2
19 (33.9)	Grade 3
2 (3.6)	Grade not assessed ^2,3^
	Histological subtype
47 (83.9)	NST
7 (12.5)	Lobular
1 (1.8)	Mixed
0	Other
1 (1.8)	Missing ^2^
	Laterality
31 (55.4)	Left
25 (44.6)	Right
0	Bilateral
	Neoadjuvant chemotherapy
25 (44.6)	Yes
24 (96.0)	Anthracycline
1 (4.0)	Non-anthracycline
31 (55.4)	No
	Pathologic tumor size
15 (26.8)	T1 (T ≤ 20 mm)
26 (46.4)	T2 (20 mm < T ≤ 50 mm)
14 (25.0)	T3 (T > 50 mm)
1 (1.8)	Missing ^2^
	Pathologic N stage
5 (8.9)	N0 ^1^
31 (55.4)	N1 (1–3)
16 (28.6)	N2 (4–9)
4 (7.1)	N3 (>10)
	Type of surgery
38 (67.9)	Conservative
17 (30.4)	Radical mastectomy
1 (1.8)	Other ^2^
	Type of axillary intervention
33 (58.9)	Axillary LN dissection
21 (37.5)	Sentinel LN biopsy
2 (3.6)	Target axillary dissection
	Adjuvant chemotherapy
28 (50.0)	Yes
21 (75.0)	Anthracycline
7 (25.0)	Non-anthracycline
28 (50.0)	No
	Radiotherapy
55 (98.2)	Yes
1 (1.8)	No
	Endocrine therapy
34 (60.7)	Anastrozole
15 (26.8)	Tamoxifen
6 (10.7)	Exemestane
1 (1.8)	Letrozole
14.5	Median abemaciclib follow-up (months)

^1^ Patients who are pathologic N0 were N+ based on clinical staging. ^2^ One patient had a previous diagnosis of left breast cancer in 2012. Currently, she presented with axillary recurrence and underwent targeted axillary dissection. ^3^ One patient was initially diagnosed in a private institution, and biopsy results were not available.

**Table 2:** Abemaciclib maintenance.

	Maintenance at 3 Months N (%)	Maintenance at 6 Months N (%)	Maintenance at 12 Months N (%)	Maintenance at 24 Months N (%)
On treatment at time-point	56 (100)	48 (85.7)	40 (71.4)	8 (14.3)
Ongoing treatment	0	8 (14.3)	16 (28.6)	48 (85.7)
Discontinuation	0	0	0	0

**Table 3:** Treatment-related toxicity profile and management strategies.

	Neutropenia	Anemia	Diarrhea	Fatigue	Nausea	Abdominal Pain
Any Grade N (%)	50 (89.3)	43 (76.8)	40 (71.4)	31 (55.4)	21 (37.5)	19 (33.9)
Grade 1–2 N (%)	42 (75.0)	41 (73.2)	39 (69.6)	31 (55.4)	21 (37.5)	19 (33.9)
Grade 3 N (%)	8 (14.3)	2 (3.6)	1 (1.8)	0 (0)	0 (0)	0 (0)
Time to onset, median, days (IQR)	28 (18–54)	41 (27–76)	31 (18–112)	42 (27–112)	18 (14–107)	27 (14–167)
Dose reduction ^1^ N (%)	3 (5.4)	3 (5.4)	13 (23.2)	5 (8.9)	3 (5.4)	5 (8.9)
Temporary interruption ^2^ N (%)	5 (8.9)	2 (3.6)	3 (5.4)	0 (0)	1 (1.8)	1 (1.8)

^1^ In some patients, two AEs were considered as it was not possible to determine which was the primary cause of dose reduction; additionally, the underlying cause of second dose reductions could be different from the first. ^2^ Two temporary treatment interruptions were attributed to other causes and were not included in this table.

**Table 4:** Temporal distribution of adverse events according to time from treatment initiation.

	Diarrhea N (%)	Neutropenia N (%)	Nausea N (%)	Abdominal Pain N (%)	Fatigue N (%)	Anemia N (%)
<3 months	28 (70)	44 (88)	14 (66.7)	11 (57.9)	23 (74.2)	36 (83.7)
3–6 months	7 (17.5)	4 (8)	5 (23.8)	4 (21.1)	3 (9.7)	3 (7)
>6 months	5 (12.5)	2 (4)	2 (9.5)	4 (21.1)	5 (16.1)	4 (9.3)

## Data Availability

The data presented in this study are available on request from the corresponding author due to privacy and ethical reasons.

## Abbreviations

The following abbreviations are used in this manuscript:

GCO	The Global Cancer Observatory
HR	Hormone Receptor
ER	Estrogen Receptor
PR	Progesterone Receptor
HER2	Human Epidermal Growth Factor Receptor 2
LN	Lymph Nodes
ET	Endocrine Therapy
EBC	Early-breast Cancer
CDK	Cyclin-dependent kinases
IDFS	Invasive Disease-free Survival
DRFS	Distant Relapse-free Survival
CI	Confidence Interval
OS	Overall Survival
pALNs	Pathologic Axillary Lymph Nodes
AEs	Adverse Events
STROBE	Strengthening the Reporting of Observational Studies in Epidemiology
CTCAEv5	Common Terminology Criteria for Adverse Events version 5.0
IQR	Interquartile Range
NST	No Special Type
PS	Performance Status
